# Hot Water as a Beverage

**Published:** 1883-06

**Authors:** 


### HOT WATER AS A BEVERAGE.

It has become quite the fashion of late to use hot water as a beverage, and, as with all agents employed in this way, there is great danger of running the thing to an extreme, and thus do harm rather than good. It is in consequence of this that we find conflicting accounts of results, particularly in the lay press, and it is our duty as medical journalists to make the subject clear if possible. First, we should start upon the physiological foundation that agents taken into the stomach at a temperature varying from 100° F. are not in the most favorable condition to digestion, and that excessive extremes from this point, not only retard this process, but sometimes set up a serious indigestion or perhaps an acute gastritis which may become chronic.

To obtain the therapeutic powers of hot water, it is important that it be taken as hot as it can be borne, and at a time when there is no food in the stomach. This point should be imperatively urged upon patients to whom this treatment is prescribed. Not long since an article appeared in the lay press warning people against the use of water above a certain temperature, upon the physiological grounds above indicated, and the advice was misleading, from the fact that the article in question did not consider the subject in all its bearings. Hot water, when taken into the stomach under these physiological restrictions, becomes one of our most valuable therapeutic means, and will relieve many an indigestion, gastritis, constipation, etc.—*N. Y. Medical Times.*

